# Interaction between iron and dihydromyricetin extracted from vine tea

**DOI:** 10.1002/fsn3.1876

**Published:** 2020-09-13

**Authors:** Liling Wang, Yuchuan Qin, Yanbin Wang, Yifeng Zhou, Bentong Liu

**Affiliations:** ^1^ Zhejiang Academy of Forestry Hangzhou China; ^2^ School of Biological and Chemical Engineering Zhejiang University of Science and Technology Hangzhou China

**Keywords:** dihydromyricetin (DMY), interaction, iron ion, solubility, stability

## Abstract

In this research, the interaction between dihydromyricetin (DMY) obtained from vine tea and iron ions (Fe (II) and Fe (III)) was investigated at pH 3.0, 5.0, and 7.0 with UV absorption and fluorescence quenching spectroscopy. The effects of DMY on the stability and solubility of iron ion were also studied. The results showed the presence of iron ions changed the UV absorption spectra of DMY at the experimental pH values. And the fluorescence spectra showed that iron ion had enhanced fluorescence effect on DMY. In addition, DMY was capable of protecting Fe (II) from being oxidized and improving the solubility of Fe (III).

## Graphical abstract

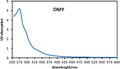

## 1 INTRODUCTION

Dihydromyricetin (3, 5, 7, 3', 4', 5'‐hexahydro‐2, 3 dihydroflavanol, or DMY), as a natural source of flavonoid, is a polyphenolic hydroxy dihydroflavanol and widely found in the genus Grapevine of the Snakeraceae. The content of DMY in vine tea is as high as 37.4% ~ 40% (Wang, Zhou, et al., 2019). Xin et al. (2013) showed that DMY had good thermal stability, but irreversible oxidation reaction would occur with the increase of temperature over 100 ℃. DMY is stable under slightly acidic conditions (Ruan, Yu, Fu, & Zhu, 2005). Moreover, as a special kind of flavonoid, DMY not only has the general properties of flavonoids, such as liver protection, antioxidation, and antibacteria, but also has the effect of inhibiting tumor activity including liver cancer (Zhang et al., 2014), breast cancer (Sun et al., 2017), and ovarian cancer (Wang et al., 2019), and has the improvement effect on hypertension (Li et al., 2017), hyperlipidemia (Wan, Jiang, Sun, Xu, & Xiao, 2017), and abnormal blood glucose (He et al., 2019). In particular, recent studies have found that DMY also has good neuroprotective activities against Alzheimer's disease (AD) (Sun et al., 2019), Parkinson's disease (PD) (Ren, Zhao, Cao, & Zhen, 2017), alcohol addiction (Liang & Olsen, 2012), and depression (Guan et al., 2019). Therefore, DMY is suitable for the research and development of food additives, antibacterial agents, and healthcare drugs, which has a broad development prospect in the food industry and the field of medicine.

Iron deficiency is a global nutritional deficiency disease (Stoltzfus, 2003), which may lead to other related diseases, such as anemia, glossitis, angular stomatitis, koilonychia, blue sclera, and esophageal webbing (Mehta, 1988). The high rate of iron deficiency anemia is mainly due to insufficient iron intake and low bioavailability from diet (Tripathi & Platel, 2011). Divalent iron (Fe (II)) and trivalent iron (Fe (III)) are the main forms existing in nature, and Fe (II) is more easily absorbed by the human body than Fe (III). The lower solubility of Fe (III) under weak acid and neutral conditions limits its absorption in the small intestine. DMY, as a kind of phenolic substance, has the common character of chelating metal ions. Under physiological conditions, the complexes of DMY with Fe (II) are formed which involve five‐member ring stably formed adjacent hydroxyl groups in DMY to iron atoms. And the formed complexes will maintain iron solubility and promote the absorption of dietary iron in the body (Li et al., 2016). Studies have shown that the metal‐binding ability of plant polyphenols can effectively protect Fe (II) autoxidation and improve the solubility of Fe (III) and its absorption in the small intestine. In addition, due to the interaction between DMY and iron, the color of DMY changed from light yellow to black, which also improved the stability of DMY (Guo, Lin, Li, Zhou, & Huang, 2005). DMY can form a stable complex with iron ions and is applied to food additives (Li et al., 2016), which has an important impact on the regulation of iron metabolism in the body. In this study, the interaction mechanism between DMY and iron ions at different pH values was studied by UV and fluorescence spectra, and the influence of DMY on the stability of Fe (II) ions and the solubility of Fe (III) ions was also studied.

## 2 MATERIALS AND METHODS

### 2.1 Plant material and reagents

DMY extract from vine tea was prepared in our laboratory. The vine tea was ground and sieved (40 mesh). The powder was mixed with 60% ethanol solution (pH 1.0) at a solid–liquid ratio of 1:25 (g/mL), and ultrasonic treatment was performed at 50 ℃ for 20 min. The extracted solution was collected, filtered, concentrated by rotary evaporation at 40 ℃, and determined by HPLC after constant volume. The HPLC was performed as the Wang, Zhou, et al. (2019) described. The main component was DMY with a content of 0.3965 g/g and a purity of more than 95%.

DMY standard was purchased from Shanghai Aladdin Chemical Co. LTD., China. The other chemicals including O‐phenylene, potassium chloride, sodium acetate anhydrous, FeSO_4_•7H_2_O (Fe (II)), FeCl_3_•6H_2_O (Fe (III)), sodium acetate anhydrous, hydroxylamine hydrochloride, and ascorbate were of analytical grade and purchased from Shanghai Aladdin Chemical Co. LTD., China.

### 2.2 Full wavelength scanning of DMY

The UV spectra of DMY extraction prepared in the laboratory were scanned in the range of 200–600 nm.

### 2.3 UV spectrum of mixed solution

The DMY extract was mixed with equal volumes iron ion (Fe (II) and Fe (III)) solutions of different concentrations under the corresponding pH values, so that the fixed concentration of DMY in the mixed solution was 0.2 mmol/L, and the concentrations of iron ions were 0.05, 0.07, 0.10, 0.20, 0.40, 0.60, 0.80, 1.00 mmol/L, and 1.60 mmol/L, respectively. After 1 hr of reaction equilibrium, the mixed solution was scanned by UV spectrophotometer from 200 nm to 600 nm. Under the same conditions, the spectral changes of pH values (3.5, 5.0, and 7.0) were measured.

### 2.4 Fluorescence spectrum of mixed solution

In the DMY–iron complex, the concentration of DMY was fixed at 0.2mmol/L, and the content ratios of DMY and iron ions were 4:1, 3:1, 2:1, 1:1, 1:2, 1:3, 1:4, 1:5, and 1:8, respectively. Fluorescence spectrum scanning was performed using a fluorescence marker, in which the wavelength of excitation light was 275 nm, the wavelength of emission was 300–400 nm, and the width of slit was 10 nm. Under the same conditions, the spectral changes of pH values (3.5, 5.0, and 7.0) in the four groups were determined.

### 2.5 Effect of DMY on stability of Fe (II)

The stability of Fe (II) experiment was performed in three groups. First, the volumetric ratio of ascorbic acid to Fe (II) was 2:1. Second, the ratio of DMY extract to Fe (II) was 2:1. Third, Fe (SO_4_)_2_ solution was used as control. The three groups of solutions were stored in a 50‐mL brown volumeter flask at room temperature, and Fe (II) retention rate was determined every 24 hr by phenanthroline spectrophotometric method (Herrera, Ruiz, Aguillon, & Fehrmann, 2010). Briefly, the samples to be tested were successively added with 1.0 ml hydroxylamine hydrochloride (10%), 1.0 ml sodium acetate (1 mol/L), and 75 ml distilled water, which were oscillated, and then added with 10ml o‐nitrophenyl solution (0.15%) for constant volume. OD value was determined with distilled water as blank under the wavelength condition of 510 nm.

### 2.6 Effect of DMY on solubility of Fe (III)

50 mmol/L ferric chloride solution was prepared with distilled water and diluted to the following concentration gradient: 2, 4, 6, 8, 10, 12, 14, 16, 18, and 20 mmol/L. The mixed solution of DMY ‐ Fe (III) was prepared with the same final DMY concentration of 0.2 mmol/L and the iron ion final concentration of 2, 4, 6, 8, 10, 12, 14, 16, 18, and 20 mmol/L, respectively.

After being stranded at room temperature for 2 hr, the samples were centrifuged at 10,800 × g for 20 min. The supernatant was taken and added into 10‐mL stopped tube. The content of iron in the supernatant was determined by the byo‐phenanthroline method, and the solubility of Fe (III) at different concentrations and the influence of DMY extract on the solubility of Fe (III) were successively described.

## 3 RESULTS AND DISCUSSION

### 3.1 Determination of maximum absorption wavelength of DMY

**Figure 1:**
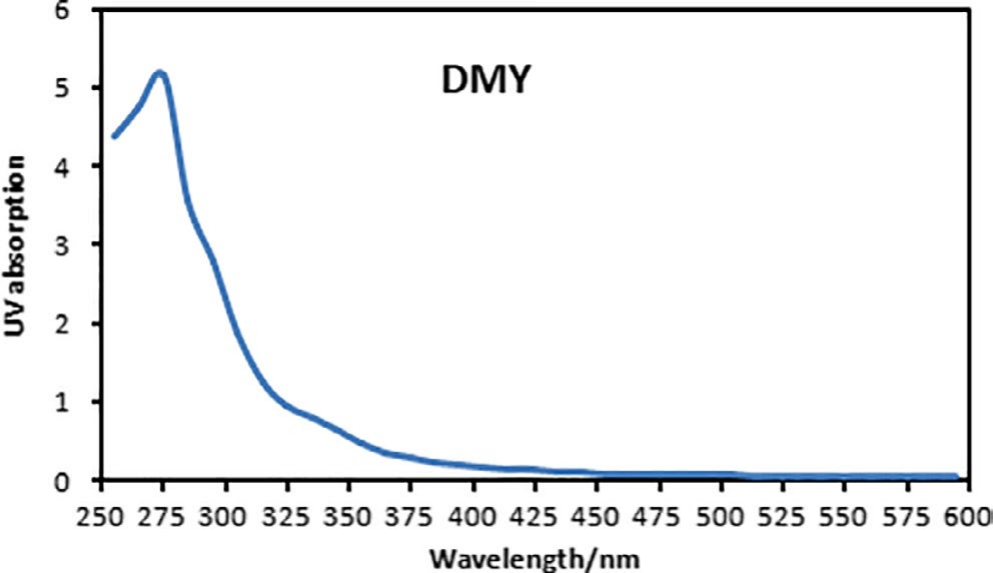
UV–Vis spectra of DMY extracted from vine tea (200–600 nm)

The extract of DMY from vine tea was diluted and scanned with UV spectrophotometer in the range of 200–600 nm. As shown in Figure 1, there are one obvious absorption peaks at the wavelength of 275 nm, of which 275 nm is the characteristic absorption peak of polyphenols (Wang, Zhou, et al., 2019). In the following experiments, the absorption wavelength of 275 nm was used to detect DMY in vine tea.

### 3.2 UV spectrometric analysis of the interaction between DMY and iron ion

**Figure 2:**
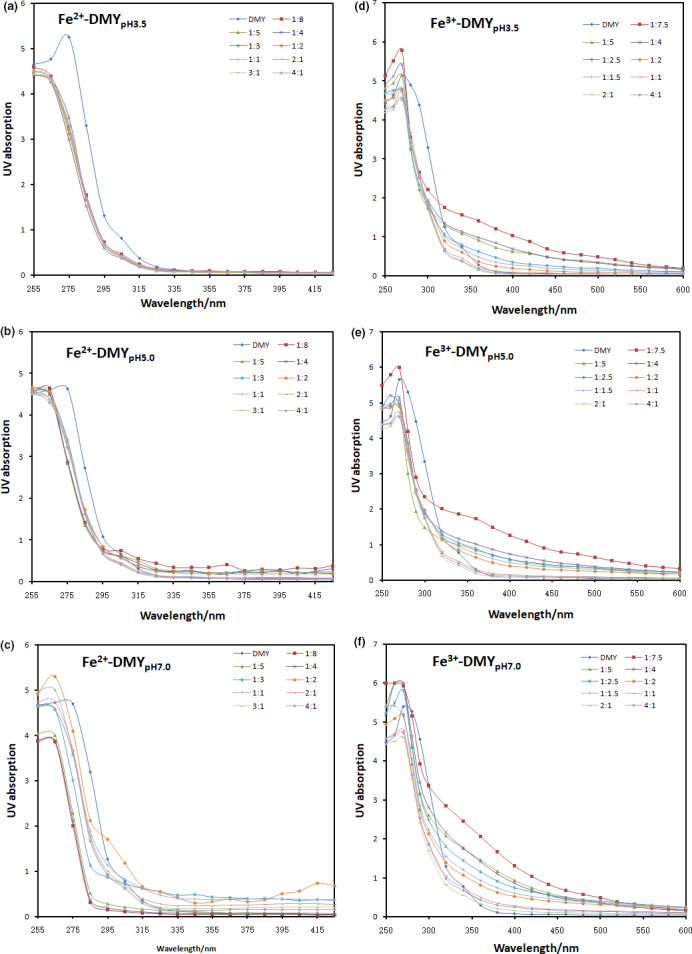
The presence of iron ions changed the UV–Vis absorption spectra of DMY at different pH values. A: DMY‐Fe (II), pH 3.5; B: DMY‐Fe (II), pH 5.0; C: DMY‐Fe (II), pH 7.0; D: DMY‐Fe (III), pH 3.5; E: DMY‐Fe (III), pH 5.0; and F: DMY‐Fe (III), pH 7.0

As shown in Figure 2a, the extraction solution of DMY has the maximum absorption at 275 nm at pH 3.5, the addition of Fe (II) ions has an obvious effect on the UV spectrum of the extraction solution of DMY, and the absorbance at 275 nm gradually decreases and blue shift occurs (Figure 2a). At pH 5.0, Fe (II) ion also had a slight effect on the absorption spectrum of DMY extract. Although the absorbance height remained unchanged, the absorption peak there appeared blue shift (Figure 2b). At pH 7.0, the blue shift of the DMY extract was caused by the addition of iron ions, and the peak strength of the absorption spectrum also changed with the difference in the concentration ratio of Fe (II) and DMY (Figure 2c). When the ratio of DMY to Fe (II) was 1:2, the absorption peak value increased significantly. When the matter ratio was 1:4 to 1:5, the absorption peak strength decreased significantly. These results indicate that iron can interacts with DMY, so the UV spectrum changes, which is in accordance with reported literature: due to the interaction between proteins and NiLs complex, the UV absorption wavelength is redshifted and the peak strength is increased (Seth et al., 2016).

Fe (III) ion also had a significant influence on the UV spectrum of DMY extract. With the increase of the concentration of iron ion, the absorbance at the wavelength of 275 nm gradually changed, and the increase was much greater than that of Fe (II) on the absorption spectrum of the extract. At pH 3.5, the absorption peak strength is higher than DMY when the mass ratio of DMY to Fe (III) is greater than 1:4, Otherwise, decreases with the decrease of Fe (III) (Figure 2d). At pH 5.0, the absorption strength reached maximum when the molality ratio of DMY to Fe (III) is 1:7.5, and the strength of absorption peak decreases gradually with the decrease of Fe (III) solution concentration (Figure 2e). At pH 7.0, the addition of Fe (III) ions results in a slightly blue shifted in the wavelength of absorption peak, and the peak's intensity varies in heights due to different proportions (Figure 2f). Generally, the higher the Fe (III) concentration, the greater the peak strength. These results indicated that ferric ion also had a significant effect on DMY, and they formed a complex that changed the wavelength of ultraviolet absorption peak, and the absorption peak size was also different due to the residual DMY in the solution caused by different proportions.

### 3.3 Fluorescence spectrometric analysis of the interaction between DMY and iron ion

Fluorescence quenching is an important means to investigate the interaction between fluorescent substances and quench agents (Zhang et al., 2011). DMY does not fluoresce, while the addition of iron ions forms chelate with DMY, causing the fluorescence of the mixed solution.

**Figure 3:**
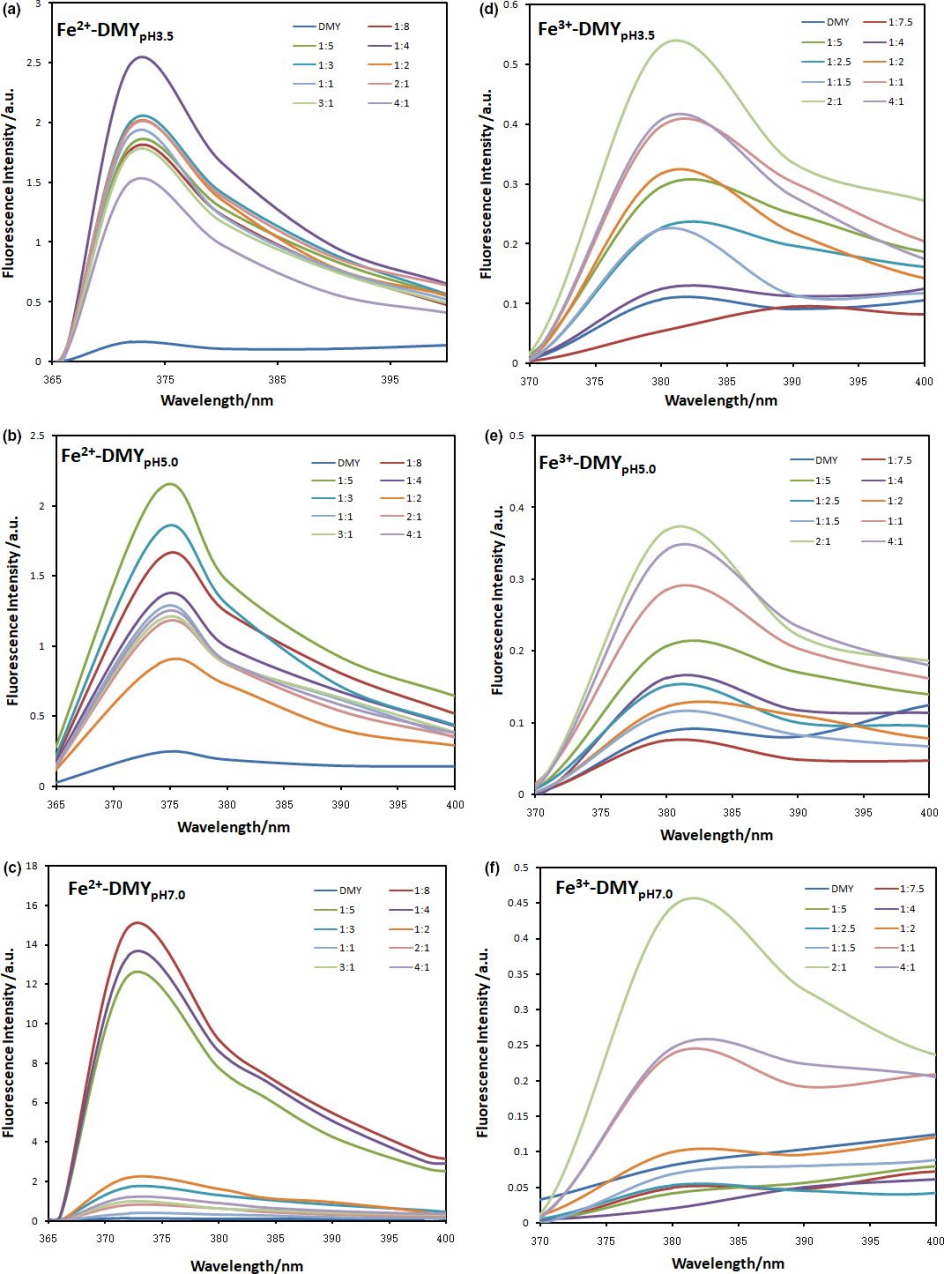
The presence of iron ions changed the fluorescence spectra of DMY at different pH values. A: DMY‐Fe (II), pH 3.5; B: DMY‐Fe (II), pH 5.0; C: DMY‐Fe (II), pH 7.0; D: DMY‐Fe (III), pH 3.5; E: DMY‐Fe (III), pH 5.0; and F: DMY‐Fe (III), pH 7.0

Figure 3 shows the fluorescence spectra of Fe (II) and Fe (III) ions with DMY at pH 3.5, 5.0, and 7.0. When the excitation wavelength was 275 nm, the maximum emission peak of DMY and Fe (II) was 375 nm and that of Fe (III) was 380 nm. With the increase of iron ions, the fluorescence intensity of the solution changed, indicating that iron ions changed the structure of DMY. This may be due to the formation of complexes between iron ions and DMY, and hydroxyl groups on both DMY A and B rings are directly involved in the chelation of metal ions (Li et al., 2016). With the increase of pH value, the fluorescence intensity of DMY solution with Fe (II) ion increased from 2.5 to 15. This is because the structure of DMY changes with the pH variation, while DMY is more stable under slightly acid pH condition (Ruan et al., 2005). After the addition of Fe (III) ion, the fluorescence intensity of DMY did not increase as much as that of Fe (II) with pH change, but both showed enhanced effects. At pH 3.5, 5.0, and 7.0, the fluorescence intensity was the strongest when the amount of DMY and Fe (III) was 2:1 (Figure 3d, e, f).

### 3.4 Effect of DMY on stability of Fe (II)

**Figure 4:**
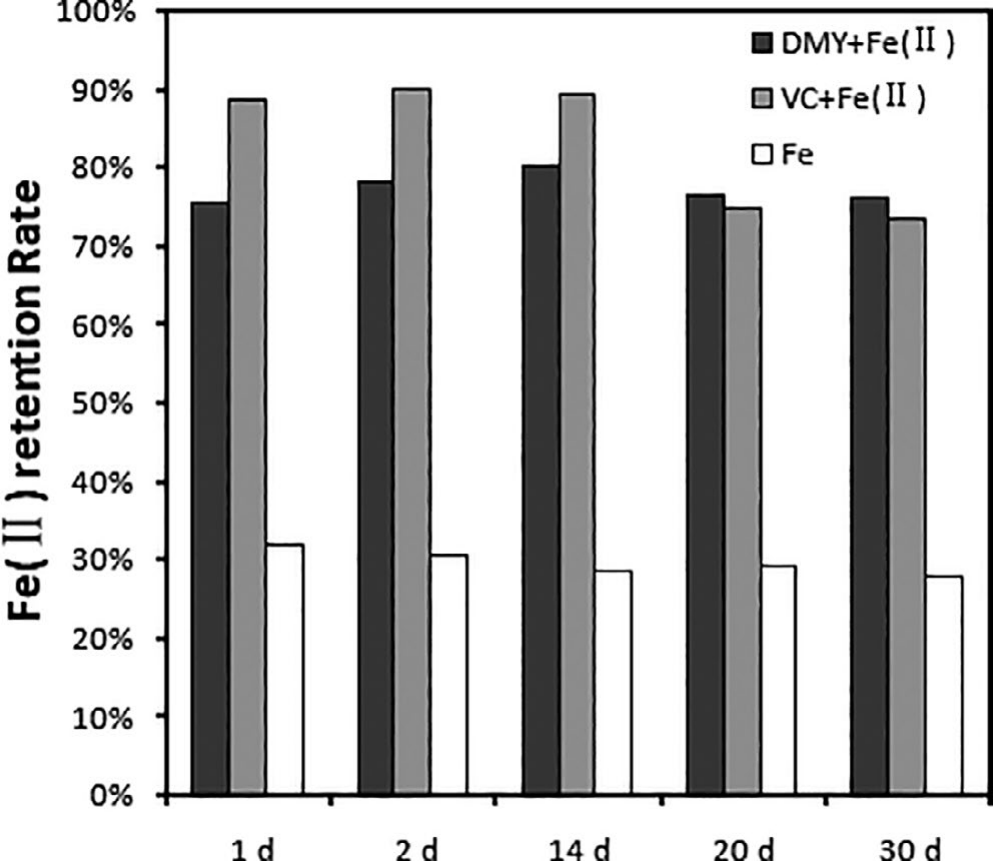
Effect of DMY extracted from vine tea and VC on stability of Fe (II)

The antioxidant is one of the main functions of DMY in vine tea (Zhang et al., 2003). Iron in nature occurs mainly in the form of Fe (III), since Fe (II) is easily oxidized to Fe (III) in air (Ilbert & Bonnefoy, 2013). Ascorbic acid and its derivatives are commonly used to protect iron ion oxidation. So, in this study, ascorbic acid was used as a comparison to investigate the effect of DMY on the stability of Fe (II) ions. As shown in Figure 4, Fe (II) is easily oxidized without antioxidants, and Fe (II) ion content decreased by about 30% in just one day, and within one month, less than 30% of the iron ions existed in the form of Fe (II). With the addition of ascorbic acid, Fe (II) was well protected and 90% Fe ions existed in the form of Fe (II) ions at the first 14 days. Compared with ascorbic acid, DMY extract had a protective effect on Fe (II) ions, although not so better than ascorbic acid at the beginning. However, DMY had better antioxidant capacity than ascorbic acid for the long‐term storage, and only 20% of the iron was oxidized after 30 days (Figure 4). Thus, it was further verified that DMY could act as an antioxidant to protect Fe (II) ions from being oxidized, so that iron ions could not participate in Fenton reaction and not react with oxygen, changing the REDOX potential and thus inhibiting Fe (II) from being oxidized to Fe (III) (Khokhar & Apenten, 2003; Winterbourn, 1995).

Studies have shown that the absorption efficiency of Fe (II) is greater than that of Fe (III). The reason is that Fe(II) can be directly received and absorbed by divalent metal transporter‐1 (DMT‐1) on the mucosa of small intestinal epithelial cells, but Fe (III) needs to be reduced by duodenal cytochrome B (DcytB) on the mucosa of intestinal epithelial cells to form Fe (II), and then transported into the cells by DMT‐1 (Griffiths, Kelly, Smith, & Cox, 2000). However, Fe (II) extremely unstable and can be oxidized or easily combined with food ingredients, thus forming complexes with low absorption utilization rate (Wang et al., 2019). Therefore, maintaining the stability of Fe (II) is of great significance for iron absorption and utilization.

### 3.5 Effect of DMY on solubility of Fe (III)

**Figure 5:**
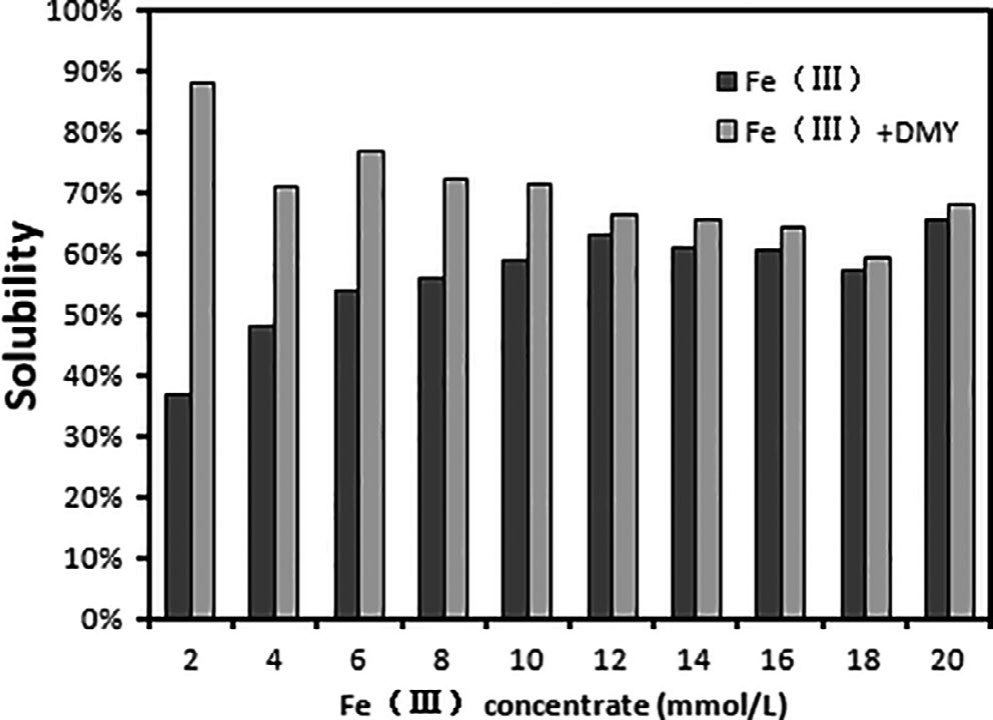
Effect of DMY extracted from vine tea on solubility of Fe (III)

The low solubility of Fe (III) under weak acid and neutral conditions limits its absorption in the small intestine. Under physiological conditions, iron can maintain its solubility by forming chelate iron, which promotes the absorption of dietary iron in the human body (Tripathi & Plate l, 2011). As show in Figure 5, when the concentration of Fe (III) in the solution was 2 mmol/L, only about 37% of Fe (III) existed in the supernatant. As the concentration increases to 12 mmol/L, the solubility of Fe (III) increased, approaching 65%. The low solubility of Fe (III) is mainly due to the formation of hydroxide compounds in the experimental environment. In addition, exposure of the solution to air also accelerates the precipitation of Fe (III) ions during the initial 2 hr of quiescence. In general, the addition of DMY enables iron ions to form chelated iron, thus improving the solubility of Fe (III) ions. When Fe (III) ion is greater than 12 mmol/L, about 70% of iron exists in the supernatant in a dissolved state. This suggested that the extract of DMY could increase the solubility of Fe (III).

All iron that the human body could absorb is soluble iron. Some dietary compounds, such as stearic acid, certain amino acids (His, Glu, Asp, and Cys), and reducing components, can enhance iron absorption (Swain, Tabatabai, & Reddy, 2002), which can bind iron, forming soluble complexes and improving iron bioavailability (Glahn & Van Campen, 1997; Storcksdieck, Bonsmann, & Hurrel, 2007). In our study, we found DMY can bind iron and increases iron solubility, which are of great help to improve the biological utilization of iron.

## 4 CONCLUSIONS

In this study, the interaction between the DMY extract and Fe (II)/Fe (III) at different pH values was studied by UV and fluorescence spectrometry. The UV spectrum showed that iron ions could affect the UV spectrum of DMY and had pH‐dependent behavior. The fluorescence spectra showed that iron can chelate DMY, and the fluorescence enhancement efficiency was changed depending on the pH. DMY extract can interact with Fe (II)/Fe (III) and can form a soluble complex under a certain substance ratio. Moreover, DMY extract has a good protective effect on the stability of Fe (II) ions and inhibits the oxidation of Fe (II) to Fe (III). The solubility of Fe (III) ions in solution is varied with different concentrations, and the solubility is lower if the concentration is low. And DMY can effectively improve the solubility of Fe (III) ions. On the whole, the DMY extract from vine tea showed strong reducing ability, high safety, and good stability. So it is suitable for the research and development of food additives and healthcare drugs, which has a broad development prospect in the food industry and the field of medicine.

## CONFLICT OF INTEREST

The authors declare that there is no conflict of interest.
